# Editorial: Insights in extreme microbiology: 2023

**DOI:** 10.3389/fmicb.2024.1473691

**Published:** 2024-08-23

**Authors:** Andreas Teske, Philippe M. Oger

**Affiliations:** ^1^Department of Earth, Marine and Environmental Sciences, University of North Carolina at Chapel Hill, Chapel Hill, NC, United States; ^2^INSA de Lyon, UMR5240 CNRS, Universite Claude Bernard Lyon 1, Villeurbanne, France

**Keywords:** editorial, extremophiles, Research Topic, *Deinococcus*, diversity

Just before the fall semester appears on the academic event horizon, the Research Topic “*Insights in extreme microbiology: 2023*” collects some interesting studies of 2023 and presents them to the audience of 2024. Fittingly for summer heat waves, this Research Topic is dominated by the theme of desiccation-, heat-, and radiation-resistant bacteria, which account for 3 of 8 papers. Two contributions focus on cell growth-inhibiting compounds from thermophiles and marine fungi, and the Research Topic is rounded off by diverse studies on microbial cesium resistance, extreme pressure tolerance, and cold-adapted Antarctic sediment communities. The studies were contributed by research groups based in China and Japan (2 each), Germany, Poland, the USA and Korea (1 each), and thus continue the trend toward geographical diversification of microbiological research, a trend that was noted in previous “*Insights in extreme microbiology*” editorials. Readers from all walks of (microbial) life should find something rewarding in the 2023 Research Topic.

While piezophilic bacteria and archaea receive much attention for their ability to thrive under extreme pressures, mechanisms of pressure tolerance and pressure adaptations in non-piezophiles are just as relevant. The study by Malas et al. uses the non-piezophilic sulfur- and metal-reducing bacterium *Shewanella oneidensis* to investigate gene expression in response to transient pressure in a high-pressure culturing system; upregulated genes include those for arginine biosynthesis, membrane reconfiguration, cold shock protein synthesis, and antioxidant defense. This study shows that non-piezophiles have adaptive capabilities that allow them to survive extreme pressures of the deep sea or dense planetary atmospheres.

New bacterial substances with antimicrobial properties provide alternatives to antibiotics. Szadkowska et al. investigate an endolysin from the extremophilic bacterium *Thermus thermophilus*, which shows similarities to previously characterized thermostable lysins (amidases) from *Thermus scotoductus* phages. This lysin turned out to be effective against other thermophiles, but only moderately so against mesophilic bacteria. In an effort to enhance its applicability, the lysin protein was searched for regions with potential activity against mesophilic bacteria. A highly positively charged peptide was identified and synthesized that turned out to depolarize bacterial membranes after binding to lipoteichoic acid, a major cell wall constituent of gram-positive bacteria, and to lipopolysaccharides on the outer membrane of gram-negative bacteria. This wide-ranging spectrum of microbial targets suggests considerable potential for lysins and lysin-derived peptides as antimicrobial agents.

To elucidate the physiological mechanism of bacterial cesium tolerance, Kojima et al. engineered a Cs^+^-resistant *E. coli* strain that tolerates several hundred millimolar of Cesium chloride in solution, and maintains ribosomal stability until 700 mM. Interestingly, this tolerance did not rely on any mutations in the high-affinity Cs^+^/H^+^ antiporter system (inserted into *E.coli* from a *Microbacterium* strain), but manifested itself in numerous single nucleotide mutations across the host genome. Of the affected genes, those encoding ribosomal modification enzymes, phage lysis regulators, and flagellar base proteins turned out to be significant for Cs^+^ resistance. Thus, cesium resistance in this *E. coli* strain appears to be primarily influenced by posttranslational protein modifications. The results have strong implications for microbially enhanced recovery of radioactive Cs^+^ from nuclear wastewater.

The search for novel antibacterial and cytostatic molecules motivated the detailed study of fungal polyketides by Cai et al., who surveyed the molecular structures of candidate compounds from a marine fungus (*Talaromyces flavus*) isolated from mangrove roots. Among multiple previously known compounds, the catch yielded three new lactones, two new polyenes, and one meroterpenoid. The study serves as a reminder that a single fungal species or isolate can produce a wide range of structurally diverse bioactive compounds.

A review by Khan et al. is discussing radiation-resistant and desiccation-resistant bacteria, and their physiological strategies of evading the stress of reactive oxygen species (ROS) intermediates. Generally, radiation-, and desiccation-resistant bacteria from arid, solar irradiation-exposed regions can regulate ROS radicals better than bacteria from non-arid regions that are not subject to evolutionary pressure from radiation exposure and low water activity. The authors discuss the linkages between evolved antioxidant systems, ROS stress, and the pervasive influence of radiation and desiccation, and examine how these factors translate into the origin, evolutionary diversity, and ecology of radiation-adapted bacteria. A brief survey of their applied and biotechnological potential concludes this contribution.

The model organism of radiation microbiology, *Deinococcus radiodurans*, and its sister species within the genus *Deinococcus grandis*, have been studied from multiple physiological and genomic angles to understand their extraordinary resistance to radiation levels that are fatal to the vast majority of organisms. To enhance the genetic tool box for *Deinococcus* studies, Sakai et al. have developed a new host-vector system for *Deinococcus grandis* that allows to introduce and to retain expression plasmids. They also developed a new plasmid where luciferase is used to examine gene expression via ultraviolet-C light irradiation.

Since the field of *Deinococcus* studies has now reached the stage where multiple wild types and genomically engineered test strains are available, a sure grasp of their genomic differences and commonalities is essential to ensure that studies performed with different model strains are mutually compatible, and are not bedeviled by single nucleotide variations, insertions and frameshift mutations, insertions and deletions. Jeong et al. perform a detailed comparison of two publicly available reference strains, *D. radiodurans* ATCC BAA-816 and ATCC 13939. Revised gene annotations account for gene fusions, and reconciliate the lengths of novel protein-coding genes, and refine the functional categorization of well-known genes. Structural variations resulting from sequence insertions highlight considerable genomic plasticity within *Deinococcus*.

The final contribution of this Research Topic returns from the survival artists that inhabit the radiation-exposed, heat-scorched and ROS-afflicted surface biosphere to a much more microbe-friendly world, cold anoxic marine sediments and their anaerobic microbial communities. Wunder et al. study marine sediments of the Antarctic peninsula that use manganese oxides as electron acceptor, and identify various branches of the Desulfuromonadales—a versatile order-level group within the Deltaproteobacteria—as the principal heterotrophic catalysts of this process. Since Antarctic sediments are impacted by gradual warming, the microbial ecology of Antarctic environments—and its preferred mode of organic matter remineralization—needs to be established in time before this baseline becomes unrecoverable.

Taken together, the wide range of themes from a diverse community of researchers show that studies of extremophilic microorganisms and extreme habitats are thriving, and provide surprises and new research angles that even the editors of this Research Topic could not have foreseen.

